# The determinants of periorbital skin ageing in participants of a melanoma case–control study in the U.K.

**DOI:** 10.1111/j.1365-2133.2011.10536.x

**Published:** 2011-11

**Authors:** M Suppa, F Elliott, JS Mikeljevic, Y Mukasa, M Chan, S Leake, B Karpavicius, S Haynes, E Bakker, K Peris, JH Barrett, DT Bishop, JA Newton Bishop

**Affiliations:** * Section of Epidemiology and Biostatistics, Leeds Cancer Research UK Centre, Leeds Institute of Molecular Medicine, University of LeedsLeeds, U.K.; † Department of Dermatology, University of L’AquilaL’Aquila, Italy; ‡ Department of Dermatology, Leeds Teaching Hospitals TrustLeeds, U.K.; § Laboratory for Diagnostic Genome Analyses, Center for Human and Clinical Genetics, Leiden University Medical CenterLeiden, the Netherlands

## Abstract

**Background:**

Skin ageing is said to be caused by multiple factors. The relationship with sun exposure is of particular interest because the detrimental cutaneous effects of the sun may be a strong motivator to sun protection. We report a study of skin ageing in participants of an epidemiological study of melanoma.

**Objectives:**

To determine the predictors of periorbital cutaneous ageing and whether it could be used as an objective marker of sun exposure.

**Methods:**

Photographs of the periorbital skin in 1341 participants were graded for wrinkles, degree of vascularity and blotchy pigmentation and the resultant data assessed in relation to reported sun exposure, sunscreen use, body mass index (BMI), smoking and the melanocortin 1 receptor (*MC1R*) gene status. Data were analysed using proportional odds regression.

**Results:**

Wrinkling was associated with age and heavy smoking. Use of higher sun-protection factor sunscreen was protective (*P*=0·01). Age, male sex, *MC1R* variants (‘r’, *P*=0·01; ‘R’, *P*=0·02), higher reported daily sun exposure (*P*=0·02), increased BMI (*P*=0·01) and smoking (*P*=0·02) were risk factors for hypervascularity. Blotchy pigmentation was associated with age, male sex, higher education and higher weekday sun exposure (*P*=0·03). More frequent sunscreen use (*P*=0·02) and *MC1R* variants (‘r’, *P*=0·03; ‘R’, *P*=0·001) were protective.

**Conclusions:**

Periorbital wrinkling is a poor biomarker of reported sun exposure. Vascularity is a better biomarker as is blotchy pigmentation, the latter in darker-skinned individuals. In summary, male sex, sun exposure, smoking, obesity and *MC1R* variants were associated with measures of cutaneous ageing. Sunscreen use showed some evidence of being protective.

Aged skin is characterized by epidermal and dermal change. The clinical signs associated with aged skin include wrinkling, elastosis, hypervascularity, irregular or blotchy pigmentation, coarseness, laxity, atrophy, dryness and itching.^1^ Hypervascularity is said to occur because capillaries of the subpapillary vascular plexus appear more visible as the epidermis becomes atrophic with age, and because of the development of dilated/elongated vessels (telangiectasia).

The above listed changes result from intrinsic ageing associated with reduced cellular proliferative capacity,^2^ but are said to be accelerated by sun exposure (photoageing). Textural changes in the skin of the hand are reported to be associated with an increased incidence of nonmelanoma skin cancers,^3^ and are assumed to be biomarkers of cumulative sun exposure. Melanocortin 1 receptor (*MC1R*) gene polymorphisms have previously been linked to sun sensitivity and low tanning response to ultraviolet radiation,^4^ and have also been described as important determinants for severe skin ageing.^5^

Cigarette smoking is also reported to play a role,^6^–^15^ and it has been suggested that this effect might be stronger in individuals with a genetic predisposition to wrinkles.^16^,^17^

Conversely, a higher body mass index (BMI) has been associated with a younger appearance of the face in general,^18^,^19^ and less wrinkling in particular.^8^,^20^ Mechanical factors such as iterative facial movements^21^ and the favoured sleeping position^22^ have also been implicated in the extent and type of wrinkling. We will henceforth refer to all these changes as cutaneous ageing, although there are multiple aetiological factors as well as chronology.

Cutaneous ageing has become increasingly of concern to many patients, and those active in health promotion have suggested that the detrimental cutaneous effects of sun exposure may prove to be a more powerful motivator to sun protection than the fear of skin cancer.^23^ We were therefore interested in understanding the predictors of these changes. A second goal was to establish whether ageing of the periorbital skin could be used as an objective marker of particular patterns of sun exposure and ultimately therefore of risk of skin cancers.

In this study we investigated the determinants of cutaneous ageing in participants in a large melanoma case–control study reported previously.^24^,^25^

## Materials and methods

### Population

A total of 797 patients with melanoma, 441 population controls ascertained by the family doctor of each case and 103 unaffected siblings of cases (sibling controls) participated as described previously.^25^ All gave written informed consent to participation in the ethically approved study.

### Data collection

Comprehensive sun-exposure data, including a life-long residence calendar were collected as previously described.^25^ Data on self-reported significant sunburns (defined as causing pain for 2 or more days), sunbed and sunscreen use were also collected. Natural hair colour at age 18 years, propensity to burn, ability to tan, skin colour of inside upper arm and freckling as a child (using the freckle chart of Gallagher *et al.*^26^) were self-reported. The highest educational level achieved was recorded as a measure of socioeconomic status. For each participant, BMI was derived from self-reported height and weight, using the formula kg m^−2^. Self-reported smoking and alcohol history (weekly units of wine, beer and spirits, a unit being defined as 10 mL of ethanol) was collected only from patients with melanoma.

### *MC1R* sequencing

Blood was collected for the extraction of germline DNA. The *MC1R* coding sequence was sequenced in 1130 participants (ABI Dye Terminator v1.1; Applied Biosystems, Warrington, U.K.), as inherited variants in this gene are associated with fair skin and susceptibility to sunburn/damage.^4^

### Photographs and grading systems

**Fig 1:**
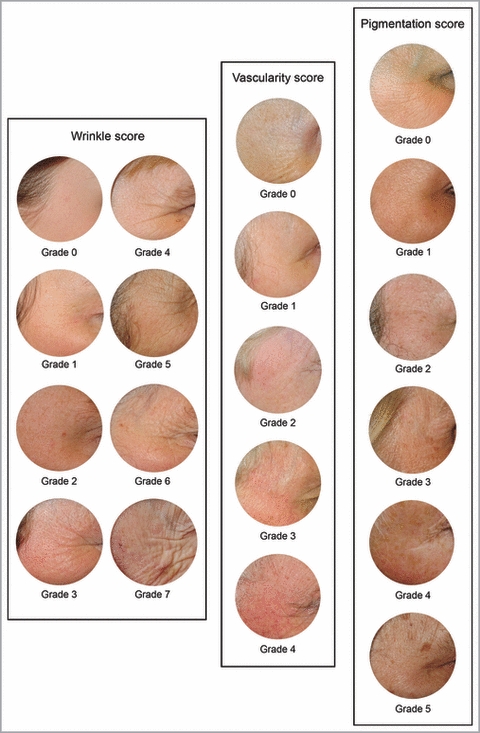
Photographic examples of the three skin ageing scores (wrinkle, vascularity and pigmentation score).

Participants were examined by trained research nurses, who recorded eye colour and photographed the right periorbital region at rest using a Canon Digital Ixus 300 camera (Canon, Reigate, U.K.), in standardized conditions (the camera was held 0·25 m away from the patient and was in macro mode). A circular laminated template, giving exposure of skin 5 cm in diameter, placed lateral to the right eye was used (Fig. 1). Three grading systems were created in order to score the photographs for three features of skin ageing: wrinkles, vascularity and blotchy pigmentation (Table 1). The pigmentation patterns observed were grouped as follows: (i) macular ill-defined pigment which is usually referred to as the blotchy pigmentation of ageing but which is difficult to distinguish from freckles; thus all macular pigment was grouped as ‘blotches’; (ii) larger more defined pigmented lesions typical of a solar lentigo; flat seborrhoeic warts are commonly clinically indistinguishable and therefore they were also categorized as solar lentigines; we will refer to these as ‘lentigines’.

**Table 1:** Grading definition for the three measures of skin ageing

Scale	Grade	Definition
Wrinkles	0	No wrinkles at all. No linear markings
	1	Just discernable linear markings
	2	1 deeper marking or several very shallow linear markings
	3	2 or 3 deeper linear markings or very many superficial
	4	4 or more deeper markings or fewer very deep
	5	4 or more deeper markings and superficial cross-hatching
	6	4 or more deeper markings and marked cross-hatching
	7	4 or more deeper markings and gross cross-hatching
Vascularity	0	No vessels or redness at all
	1	Redness but no discernible vessels
	2	≤ 5 just discernible telangiectasia
	3	> 5 just discernible telangiectasia or ≤ 5 evident telangiectasia
	4	> 5 evident telangiectasia
Pigmentation	0	No pigmentation at all
	1	≤ 2 blotches
	2	> 2 blotches or ≤ 2 lentigines (not simultaneously)
	3	Nonconfluent blotches and ≤ 2 lentigines
	4	Confluence of blotches (irrespective of lentigines) or 3–6 lentigines (irrespective of blotches)
	5	> 6 lentigines

Pigmented naevi and melasma were not included in this evaluation. The pigmentation score was therefore a summation of the number of blotches and lentigines observed (Table 1).

The digital photographs were assessed for the three measures of ageing by a postresidency dermatologist (M.S.). For internal consistency, the scoring system was tested by repeat scoring to develop a reproducible system. One hundred photos were assessed independently by an equally experienced dermatology registrar (J.S.M.) to assess interobserver agreement, and the level of agreement was formally assessed.

### Statistical analysis

All continuous measures displayed a skewed distribution and were therefore divided into tertiles/quartiles based on the overall distribution. BMI was categorized according to the World Health Organization’s classification.^27^*MC1R* alleles were classified as ‘R’, ‘r’ or neither, where ‘R’ variants are strongly, and ‘r’ weakly associated with red hair.^4^ Cigarette smoking was quantified as ‘pack-years’, a commonly used measure of smoking, which is calculated by multiplying the average number of packs (20 cigarettes) smoked per day by the number of years the person has smoked.^28^ For example, 1 pack-year is equal to smoking 20 cigarettes (one pack) per day for 1 year or 10 cigarettes (half pack) per day for 2 years and so on. A proxy measure for sun sensitivity was derived by applying a factor analysis to correlated phenotypic variables, as previously described.^25^ Spearman’s correlation coefficients were estimated to examine correlation between all continuous or ordered categorical variables. As the three skin ageing gradings were ordinal categorical variables, the Fleiss–Cohen quadratic-weighted kappa (κ) statistic was chosen to calculate intraobserver and interobserver agreement, as previously reported.^29^ As phenotype was measured on an ordinal scale, proportional odds regression models were used to determine predictors of wrinkles, vascularity and pigmentation. Further information is given in Data S1 (see Supporting Information). Models including ordered categorical variables were analysed as a test for linear trend. ‘Simple’ models were estimated whereby each factor was assessed as a predictor of each phenotype, adjusted for the ‘nuisance’ variables: age, sex and highest educational level. Melanoma case–control status was also adjusted for in the models. Factors significant at the arbitrary 5% level in the simple models were then entered together into other, more ‘complicated’ models, to identify independent predictors. The analysis was carried out using the STATA version 10, 2007 (StataCorp., College Station, TX, U.S.A.).

## Results

The median age at examination of the participants was 56 years and 60% were female (Table 2). Poorly defined photographs were not fully scored, thus a wrinkle score could not be determined for four participants, or vascularity and pigmentation for nine participants. The vascularity score was significantly positively correlated both with the wrinkle (ρ = 0·08, *P*=0·004) and pigmentation scores (ρ = 0·12, *P*<0·001) but the wrinkle and pigmentation scores were not correlated (ρ = 0·03, *P*=0·31) (data not shown).

**Table 2:** Descriptive statistics of the study population

	*n* (%)^a^	Median (range)
Age at examination (years)		
< 44	333 (24·8)	55·6 (19·1–87·2)
44–54	319 (23·8)	
55–65	361 (26·9)	
> 65	328 (24·5)	
Sex		
Female	810 (60·4)	–
Male	531 (39·6)	
Highest educational level		
Primary/secondary school	465 (35·2)	–
Sixth form/vocational training	561 (42·5)	
University/postgraduate	295 (22·3)	
Sun-sensitive phenotype		
No	670 (50·8)	–
Yes	608 (49·2)	
*MC1R* genotype		
−/− (wild-type)	226 (20·0)	–
r/−	259 (22·9)	
r/r	96 (8·5)	
R/−	269 (23·8)	
R/r	186 (16·5)	
R/R	94 (8·3)	
BMI		
< 25	571 (43·3)	25·8 (15·6–68·4)
25–30	508 (38·5)	
> 30	240 (18·2)	
Smoking		
Never	410 (52·8)	–
Ever	366 (47·2)	
Pack-years^b^		
0	412 (55·3)	0 (0–120)
0–15	164 (22·0)	
> 15	169 (22·7)	
Average alcohol intake (units per week)^c^		
≤ 1	195 (25·3)	6 (0–99)
2–6	205 (26·6)	
7–13	181 (23·5)	
> 14	190 (24·6)	
Sunburn – no. before age 20 years		
0	941 (72·8)	0 (0–152)
1–12	216 (16·7)	
> 12	136 (10·5)	
Sunburn – no. at or after age 20 years		
0	849 (66·4)	0 (0–240)
1–10	292 (22·9)	
> 10	137 (10·7)	
Sunburn – average no. in life		
0	647 (50·4)	0 (0–360)
1–26	318 (24·8)	
> 26	318 (24·8)	
Sunbed – ever vs. never		
Never	714 (54·2)	–
Ever	603 (45·8)	
Sunbed – no. of sessions in life		
0	714 (55·1)	0 (0–5000)
1–20	308 (23·8)	
> 20	273 (21·1)	
Sunscreen – used to avoid sunburn		
Never or hardly ever	496 (37·0)	–
Not often	447 (33·3)	
Often	398 (29·7)	
Sunscreen – used to stay in the sun longer		
Never or hardly ever	865 (64·5)	–
Not often	224 (16·7)	
Often	252 (18·8)	
Sunscreen – SPF level		
Never or hardly ever	512 (38·2)	–
SPF < 10	469 (35·0)	
SPF ≥ 10	360 (26·8)	
Wrinkle score^d^		
Grades 0 and 1	177 (13·2)	–
Grade 2	351 (26·3)	
Grade 3	415 (31·0)	
Grade 4	212 (15·9)	
Grades 5–7	182 (13·6)	
Vascularity score^d^		
Grades 0 and 1	306 (23·0)	–
Grade 2	432 (32·4)	
Grade 3	409 (30·7)	
Grade 4	185 (13·9)	
Pigmentation score^d^		
Grades 0 and 1	112 (8·4)	–
Grade 2	427 (32·1)	
Grade 3	471 (35·3)	
Grades 4 and 5	322 (24·2)	

BMI, body mass index; SPF, sun-protection factor.

a Numbers do not always total 1341 due to missing data.

b A ‘pack-year’ is defined by the formula: number of daily cigarettes × years smoked/20.

c In the U.K., an ‘alcohol unit’ is defined as 10 mL of pure alcohol (ethanol).

d Grades 0 and 1 were grouped in the three skin ageing scales as well as grades 5–7 and 4 and 5 in the wrinkle and the pigmentation scales, respectively, due to low numbers.

The intraobserver agreement was ‘almost perfect’ (κ 0·92, 0·96 and 0·92 for wrinkles, vascularity and pigmentation, respectively; *P*<0·001 for all), while the interobserver agreement ranged from ‘fair’ to ‘substantial’ (κ 0·71, 0·42 and 0·39 for wrinkles, vascularity and pigmentation, respectively; *P*<0·001 for all), as defined by Landis and Koch.^30^

Increasing age and male sex were risk factors for all three phenotypic measures of ageing (Table 3). Highest educational level attained was positively associated with pigmentation, negatively with wrinkling and not associated with vascularity.

**Table 3:** Predictors of the three skin ageing measures in proportional odds regression models adjusted for age, sex, highest educational level attained and melanoma status

	Wrinkles			Vascularity			Pigmentation		
			
	*n*	OR (95% CI)	*P-*value^a^	*n*	OR (95% CI)	*P-*value^a^	*n*	OR (95% CI)	*P-*value^a^
Age at examination^b^	1337	**2·50** (**2·26–2·76**)	**< 0·001**	1332	**1·35** (**1·23–1·48**)	**< 0·001**	1332	**1·12** (**1·03–1·23**)	**0·01**
Sex (male vs. female)^b^	1337	**1·56** (**1·28–1·89**)	**< 0·001**	1332	**7·52** (**5·99–9·45**)	**< 0·001**	1332	**1·43** (**1·17–1·74**)	**0·001**
Highest educational level^b^	1317			1312			1312		
Primary/secondary school	464	1		460	1		459	1	
Sixth form/vocational training	559	**0·71** (**0·57–0·88**)	**0·002**	557	0·84 (0·67–1·05)	0·13	559	**1·29** (**1·03–1·62**)	**0·03**
University/postgraduate	294	**0·55** (**0·42–0·72**)	**< 0·001**	295	0·92 (0·71–1·20)	0·55	294	**1·57** (**1·20–2·05**)	**0·001**
Sun-sensitive phenotype (yes vs. no)	1298	0·83 (0·67–1·01)	0·07	1292	1·05 (0·86–1·29)	0·63	1292	0·84 (0·69–1·03)	0·10
*MC1R* genotype	1110			1106			1104		
Wild-type (wt)	223	1		223	1		223	1	
r/wt	256	1·12 (0·81–1·55)	0·48	254	**1·39** (**0·99–1·94**)	**0·05**	255	0·77 (0·56–1·07)	0·13
r/r	95	1·08 (0·69–1·68)	0·75	94	1·25 (0·80–1·96)	0·33	94	**0·56** (**0·36–0·86**)	**0·01**
R/wt	263	1·01 (0·73–1·40)	0·96	261	1·09 (0·78–1·52)	0·60	260	**0·55** (**0·39–0·76**)	**< 0·001**
R/r	181	1·21 (0·85–1·74)	0·29	180	**1·45** (**1·01–2·10**)	**0·05**	179	**0·58** (**0·41–0·83**)	**0·003**
R/R	92	0·80 (0·51–1·25)	0·33	94	**1·73** (**1·10–2·72**)	**0·02**	93	1·15 (0·72–1·83)	0·55
*MC1R* genotype – grouped	1110			1106			1104		
Wild-type	223	1		223	1		223	1	
‘r’ without ‘R’	351	1·11 (0·82–1·51)	0·50	348	1·35 (0·99–1·85)	0·06	349	**0·71** (**0·52–0·96**)	**0·03**
‘R’	536	1·03 (0·78–1·38)	0·82	535	1·29 (0·97–1·73)	0·08	532	**0·63** (**0·47–0·83**)	**0·001**
BMI	1303	0·98 (0·86–1·13)	0·82	1298	**1·23** (**1·07–1·41**)	**0·003**	1298	1·01 (0·88–1·15)	0·93
Smoking (ever vs. never)^c^	758	0·97 (0·74–1·27)	0·81	758	1·16 (0·89–1·52)	0·28	756	0·87 (0·66–1·14)	0·30
Pack-years^c^	729	1·08 (0·91–1·27)	0·37	727	**1·21** (**1·02–1·43**)	**0·03**	726	0·88 (0·74–1·04)	0·13
Average alcohol intake^c^	753	0·98 (0·86–1·11)	0·71	754	0·91 (0·80–1·04)	0·16	752	0·90 (0·79–1·02)	0·11
Average daily sun exposure	1256	0·98 (0·89–1·08)	0·70	1252	**1·12** (**1·02–1·24**)	**0·02**	1252	1·06 (0·97–1·17)	0·20
Average weekday sun exposure – overall	1257	0·96 (0·88–1·06)	0·44	1252	**1·12** (**1·02–1·23**)	**0·02**	1252	1·09 (0·99–1·19)	0·07
Average weekday sun exposure – cooler months	1258	0·95 (0·87–1·05)	0·31	1253	**1·11** (**1·01–1·22**)	**0·04**	1253	1·06 (0·97–1·16)	0·22
Average weekday sun exposure – warmer months	1262	1·00 (0·92–1·10)	0·94	1257	1·09 (0·99–1·20)	0·06	1257	**1·11** (**1·01–1·22**)	**0·03**
Average weekend sun exposure – overall	1269	0·99 (0·90–1·10)	0·88	1265	1·05 (0·95–1·16)	0·35	1265	1·03 (0·94–1·14)	0·50
Average weekend sun exposure – cooler months	1272	0·95 (0·86–1·05)	0·30	1268	1·01 (0·91–1·12)	0·88	1268	1·03 (0·93–1·13)	0·62
Average weekend sun exposure – warmer months	1271	1·06 (0·97–1·17)	0·22	1267	1·06 (0·96–1·17)	0·22	1267	1·04 (0·94–1·14)	0·46
Average holiday sun exposure – overall	1270	1·04 (0·95–1·14)	0·38	1266	1·03 (0·94–1·13)	0·55	1266	1·04 (0·95–1·14)	0·39
Average holiday sun exposure – 10:00–14:00 h	1265	1·01 (0·92–1·11)	0·78	1261	1·06 (0·96–1·17)	0·23	1261	1·05 (0·96–1·15)	0·32
Average holiday sun exposure – below 45° N latitude	1270	1·02 (0·92–1·12)	0·74	1266	1·02 (0·93–1·12)	0·70	1266	1·08 (0·98–1·18)	0·13
Average holiday sun exposure – below 45° N latitude (10:00–14:00 h)	1265	1·00 (0·91–1·10)	0·99	1261	1·02 (0·93–1·12)	0·66	1261	1·09 (0·99–1·20)	0·06
Sunburn – no. before age 20 years	1272	0·87 (0·75–1·02)	0·08	1267	1·04 (0·90–1·23)	0·56	1267	0·90 (0·77–1·05)	0·19
Sunburn – no. at or after age 20 years	1257	1·07 (0·92–1·25)	0·36	1252	1·06 (0·92–1·25)	0·40	1252	0·93 (0·80–1·09)	0·37
Sunburn – average no. in life	1262	0·92 (0·81–1·04)	0·16	1257	1·05 (0·93–1·19)	0·42	1257	0·90 (0·80–1·02)	0·11
Sunbed – ever vs. never	1296	0·99 (0·80–1·24)	0·97	1291	0·96 (0·77–1·19)	0·70	1291	0·88 (0·71–1·10)	0·26
Sunbed – no. of sessions in life	1274	0·94 (0·82–1·08)	0·37	1269	0·97 (0·84–1·12)	0·66	1269	0·93 (0·81–1·07)	0·30
Sunscreen – used to avoid sunburn	1317	0·90 (0·79–1·02)	0·11	1312	0·98 (0·87–1·12)	0·81	1312	0·90 (0·79–1·03)	0·12
Sunscreen – used to stay in the sun longer	1317	0·93 (0·82–1·06)	0·29	1312	1·00 (0·88–1·14)	0·98	1312	**0·86** (**0·76–0·98**)	**0·03**
Sunscreen – SPF level	1317	**0·84** (**0·74–0·96**)	**0·01**	1312	0·99 (0·87–1·14)	0·97	1312	0·93 (0·82–1·06)	0·29

Findings significant at the 5% level are highlighted in bold. *n,* number of observations in each model; OR, odds ratio; CI, confidence interval; BMI, body mass index; SPF, sun-protection factor.

a *P*-values for trend are presented, as displayed in Table 2, except for highest educational level and *MC1R* genotypes. Sun-exposure measures were analysed as a trend across quartiles.

b Adjusted only for melanoma status.

c Adjusted only for age, sex and education.

Table 3 shows predictors of the three skin ageing scores in proportional odds regression models adjusted for age, sex, educational level attained and case–control status (‘simple’ models).

Reported use of higher sun-protection factor (SPF) sunscreen was protective for wrinkles (test for trend, *P*=0·01). There was no effect of pack-years of smoking on wrinkling when assessed as a test for trend (*P*=0·37) but there was a significant effect in heavy smokers (≥ 40 pack-years, *n*=43) when compared with nonsmokers (*n*=412) [odds ratio (OR) 1·91, 95% confidence interval (CI) 1·04–3·51, *P*=0·04]. There was no effect of lighter smoking (< 40 pack-years, *n*=290) when compared with nonsmokers (OR 0·90, 95% CI 0·68–1·19, *P*=0·47) (data not shown).

Increased BMI, smoking pack-years, and total and weekday (overall and cooler months) sun exposure were risk factors for hypervascularity, as well as the presence of the ‘R’/ ‘r’ and ‘R’/ ‘R’*MC1R* genotypes.

Greater weekday sun exposure in the warmer months was associated with increased pigmentation, while the presence of any ‘R’ or ‘r’ variant of *MC1R* was protective, as was reported use of sunscreen to stay in the sun longer (test for trend, *P*=0·03).

In order to eliminate the potential confounding effect of sex, we repeated the same analyses separately in male and female subjects and found estimates consistent with the combined analysis (data not shown). Similarly, in order to eliminate the confounding effect of melanoma status, the same analyses were performed separately in cases and controls. Estimates consistent with the combined analysis were found except for *MC1R* genotype, whose effect on vascularity could be seen almost entirely in controls (Table S1; see Supporting Information).

Pack-years of smoking was positively correlated with weekday and weekend sun exposure but negatively correlated with sunny holiday exposure. All the sunburn, sunbed and sunscreen measures, as well as highest educational level were positively correlated with holiday exposure and negatively correlated with the other sun-exposure measures (data not shown).

Factors found to be significantly associated with the three skin ageing measures in simple analyses reported in Table 3 were entered together into more complex models to identify independent predictors of skin ageing phenotypes (Table 4). Increasing age and a protective effect of use of higher SPF sunscreen remained independent predictors of wrinkling. Three models were fitted for predicting vascularity due to missing *MC1R* genotype and smoking data. The first model excluded the *MC1R* and smoking variables, to give more power to look at sun exposure as these data were available only for a subset of participants. Age, male sex, higher BMI and higher average daily sun exposure were found to be independent predictors of increased vascularity. Age, male sex, *MC1R* variants and BMI were found to be independent predictors in the second model where only smoking was excluded. Average daily sun exposure was no longer significant at the 5% level probably due to the reduced numbers, although the OR was not dissimilar. In the third model including all the significant predictors found in Table 3, male sex, BMI and pack-years were independent predictors. Overall there was evidence for male sex, age, higher BMI, average higher sun exposure, variant *MC1R* alleles and smoking as predictors of hypervascularity.

**Table 4:** Predictors of the three skin ageing measures from proportional odds regression models including the significant predictors in Table 3, age, sex, highest educational level attained and melanoma status

			Vascularity^a^						Pigmentation^b^			
				
	Wrinkles *n*=1317		Model 1 *n*=1241		Model 2 *n*=1045		Model 3 *n*=630		Model 1 *n*=1257		Model 2 *n*=1058	
						
	OR (95% CI)	*P*-value^c^	OR (95% CI)	*P*-value^c^	OR (95% CI)	*P*-value^c^	OR (95% CI)	*P*-value^c^	OR (95% CI)	*P*-value^c^	OR (95% CI)	*P*-value^c^
Age at examination	**2·39** (**2·16–2·65**)	**< 0·001**	**1·25** (**1·13–1·38**)	**< 0·001**	**1·18** (**1·06–1·32**)	**0·002**	**1·14** (**1·00–1·30**)	**0·05**	**1·11** (**1·01–1·23**)	**0·03**	1·08 (0·97–1·20)	0·15
Sex (male vs. female)	1·13 (0·91–1·39)	0·27	**6·75** (**5·27–8·65**)	**< 0·001**	**6·47** (**4·95–8·45**)	**< 0·001**	**6·63** (**4·65–9·49**)	**< 0·001**	1·23 (0·99–1·52)	0·06	**1·28** (**1·01–1·62**)	**0·04**
Highest educational level												
Primary/secondary school	1		1		1		1		1		1	
Sixth form/vocational training	0·96 (0·76–1·20)	0·72	0·83 (0·65–1·05)	0·13	0·79 (0·60–1·02)	0·07	0·76 (0·55–1·07)	0·12	**1·43** (**1·13–1·81**)	**0·003**	**1·46** (**1·13–1·89**)	**0·004**
University/postgraduate	0·85 (0·65–1·12)	0·24	1·00 (0·75–1·34)	0·99	0·96 (0·70–1·31)	0·79	1·00 (0·66–1·52)	0·99	**1·73** (**1·30–2·29**)	**< 0·001**	**1·57** (**1·16–2·13**)	**0·004**
*MC1R* genotype – grouped^d^												
Wild-type					1		1				1	
‘r’ without ‘R’					**1·53** (**1·11–2·12**)	**0·01**	1·20 (0·78–1·87)	0·41			**0·71** (**0·52–0·97**)	**0·03**
‘R’					**1·44** (**1·06–1·95**)	**0·02**	1·13 (0·76–1·67)	0·54			**0·61** (**0·46–0·82**)	**0·001**
BMI			**1·20** (**1·04–1·38**)	**0·01**	**1·26** (**1·08–1·47**)	**0·003**	**1·25** (**1·02–1·52**)	**0·03**				
Pack-years							**1·25** (**1·04–1·50**)	**0·02**				
Average daily sun exposure^e^			**1·13** (**1·02–1·24**)	**0·02**	1·10 (0·99–1·23)	0·07	1·08 (0·94–1·24)	0·26				
Average weekday sun exposure – warmer months									**1·11** (**1·01–1·22**)	**0·03**	1·10 (0·99–1·22)	0·07
Sunscreen – used to stay in the sun longer									**0·85** (**0·74–0·97**)	**0·02**	**0·85** (**0·73–0·98**)	**0·03**
Sunscreen – SPF level	**0·84** (**0·74–0·96**)	**0·01**										

Findings significant at the 5% level are highlighted in bold. *n,* number of observations in each model; OR, odds ratio; CI, confidence interval; BMI, body mass index; SPF, sun-protection factor.

a Model 1 excludes *MC1R* and smoking data, model 2 includes *MC1R* but not smoking, model 3 includes both *MC1R* and smoking.

b Model 1 excludes *MC1R*, model 2 includes *MC1R*.

c *P*-values for trend are presented, as displayed in Table 2, except for highest educational level and *MC1R* genotype.

d Models including *MC1R* genotype nongrouped rather than grouped displayed similar results.

e Models including average weekday (overall and cooler months) rather than daily sun exposure displayed similar results.

Similarly, two models were fitted for predicting blotchy pigmentation due to missing *MC1R* data. Age, higher educational levels attained, greater weekday sun exposure in warmer months and lower use of sunscreen reported by participants to allow them to stay in the sun longer were found to be independent predictors in the first model, which excluded *MC1R*. Male sex, higher educational levels attained, *MC1R* and sunscreen used to stay in the sun longer were found to be independent predictors in the second model which included all the significant predictors found in Table 3. Age and weekday exposure in warmer months were no longer significant at the 5% level and again this may be due to the reduced numbers. Overall, there was evidence for male sex, age, higher educational level, greater weekday sun exposure in warmer months, absence of variant *MC1R* alleles, and sunscreen used to stay out in the sun longer as predictors of blotchy facial pigmentation.

## Discussion

This study investigated the determinants of cutaneous ageing in individuals living in a temperate climate. The periorbital region is one of the first places on the face to show signs of ageing,^31^ not least because it is not shaded by the contour of the face or hair, and it is one of the areas most frequently considered for rejuvenation treatment.^32^

Three measures of skin ageing were assessed by evaluating digital photographs of the right periorbital skin using scoring systems developed by the authors. Two photonumeric scales for wrinkles and pigmentary changes similar to ours have been reported.^9^ Our scoring system was reproducible (intraobserver and interobserver).

Age was the only independent risk factor for periorbital wrinkling in the study population overall, although many previous studies have suggested that sun exposure is related to wrinkling. Cumulative lifetime sun exposure has been reported to increase periorbital wrinkling in particular,^33^–^36^ yet we did not see an association with reported exposure. None the less, reported high SPF sunscreen use was independently protective in our population, suggesting a role for sun exposure in wrinkling. Creasing of the periorbital skin also results from smiling and squinting^21^ and it is suggested that the repetitive contraction of underlying lateral orbicularis oculi can lead to ‘crow’s feet’ due to changes in the elastic properties of the dermis over time.^37^ It may be that we did not see a strong relationship between wrinkling in this site and sun exposure because there is too much variation due to patterns of facial movements to see a smaller effect of reported sun exposure.

Periorbital wrinkles have been reported to be associated with the duration and intensity of cigarette smoking in a number of studies.^6^–^15^ We showed evidence of an independent effect for very heavy smokers only and comparison of the study populations suggested that our sample consisted of a population with significantly fewer heavy smokers than in previous studies.^6^,^9^,^14^ For example, we had only 2% of smokers who reported 50 or more pack-years compared with 19% in the study of Kadunce *et al.*^6^ Our study therefore demonstrates that the effect of smoking on periorbital wrinkling is less clear than the literature would suggest, at least for moderate smokers, as suggested by others.^38^

The hallmark of facial ageing is volume loss, particularly in the mid face, due to atrophy and malposition of fat pads.^39^ Several authors have reported higher BMI associated with less wrinkling^8^,^20^ as well as a younger overall appearance of the face.^18^,^19^ We saw no evidence for such a relationship in our analysis and, indeed, there was no evidence in other studies, which focused exclusively on periorbital wrinkles.^14^,^40^ This may reflect the fact that periorbital wrinkles have less of an overall impact on the appearance of facial ageing compared with changes in the mid face.

In this study, hypervascularity was more persuasively related to reported sun exposure than periorbital wrinkles. It was positively correlated with average daily and weekday sun exposure, and was also associated with *MC1R* variants, known to be associated with sun sensitivity.^4^ Smoking and increased age were also associated with cutaneous hypervascularity and published data suggest that these associations probably result from relative cutaneous hypoxia.^41^,^42^ Decreased density but increased length of cutaneous capillaries were reported in elderly individuals compared with younger subjects.^43^ Heavy smoking is said to compromise the peripheral microvasculature,^44^ leading to chronic ischaemia of the dermis and the consequent development of telangiectasia as a compensatory mechanism. We also showed an association between increased BMI and periorbital hypervascularity. Obesity may conceivably increase vascularity as a result of poorer temperature control-induced persistent vasodilation, but secreted products of fat cells (adipokines) and macrophages in fat are also postulated to induce new blood vessel formation.^45^ The severity of vascular damage in the periorbital area was strongly predicted by sex in our population. Males were consistently more prone to develop hypervascularity even when controlling for all the potential confounders. As previously reported,^46^ telangiectasia may be a weaker marker of skin ageing in women due to the protective effect of oestrogens on skin microvasculature. Skin capillary blood flow was shown to increase with the oestrogen level^47^ and to decrease significantly with menopause.^48^ A protective role of physiological concentrations of oestrogens was also reported in a mouse model of skin ischaemia.^49^

Age was shown to be a significant independent risk factor for blotchy pigmentation, although this was no longer significant when the analysis was further corrected for *MC1R*, possibly due to reduced sample size. Male sex was also independently predictive which is unexplained but may reflect unmeasured differences in sun exposure or sunscreen use. Higher educational level achieved was a risk factor for blotchy pigmentation, which is also unexplained. We looked at the correlations with higher educational level achieved in order to explain this: we saw a positive correlation with sunny holiday exposure and a negative correlation with all the other patterns of sun exposure, but have not identified obvious explanations for this finding. There was reasonable evidence to suggest that blotchy pigmentation is related to sun exposure: weekday sun exposure in warmer months was a risk factor for pigmentation and sunscreen use to stay in the sun longer was significantly protective. Carriers of ‘R’ variants of *MC1R* were less likely to have a high score for pigmentation in our analysis. ‘R’ variants are associated with the ‘red hair colour phenotype’^4^ and paler skin so that this is not unexpected, although other authors have reported the contrary.^5^ As previously reported,^9^ no significant association was found between smoking and increased pigmentation. We conclude therefore that blotchy facial pigmentation is more likely to be seen in individuals without *MC1R* variants who have greater sun exposure.

One goal of this study was to establish whether ageing of the periorbital skin could be used as an objective biomarker of sun exposure. Overall, in clinic and as a potential marker in epidemiological studies therefore, increased cutaneous vascularity would appear to be the best biomarker of regular sun exposure although it would be necessary to allow for the other determinants of vascularity such as age, sex, smoking and obesity.

The strengths of the study are that this is the largest reported, and a reproducible measure was used by a blinded observer. Furthermore, the questionnaire used to collect sun exposure data was validated, internationally used and detailed.^24^,^50^–^52^ The weaknesses are that only one facial site was investigated and the use of sunglasses and eyeglasses was not recorded. Furthermore, this was not a population-based study and multiple factors were tested, therefore *P*-values should be interpreted bearing this in mind.

In conclusion, this study supports the evidence that smoking, obesity and excessive sun exposure increase the appearance of ageing of the skin, specifically in the periorbital region. The study produced some evidence that sunscreen use is protective for age-related cutaneous damage.

**What’s already known about this topic?**

- Skin ageing is determined by exposures such as sun exposure and smoking, as well as chronological age.
- Independent risk factors have been poorly studied.
- Skin cancer risk is increased by sun exposure but the complexities are such that it is difficult to understand the risk associated with different types of sun exposure. Phenotypic biomarkers of exposure are needed.

**What does this study add?**

- Periorbital wrinkling is a poor biomarker of reported sun exposure.
- Periorbital telangiectasia is a better biomarker of cumulative sun exposure, as well as blotchy pigmentation in darker-skinned individuals.
- Cigarette smoking, obesity and *MC1R* variants are associated with different measures of periorbital ageing.
- Heavy smoking is associated with wrinkling but moderate smoking less convincingly so.
- Sunscreen use shows evidence of a protective role for skin ageing.

## Supporting Information

**Data S1** Supplementary materials and methods.

**Table S1** Predictors of the three skin ageing measures in proportional odds regression models found to be significant at the 5% level in the primary analysis, stratified by melanoma status.
