# Supplementary material for: The determinants of periorbital skin ageing in participants of a melanoma case–control study in the U.K
Source: Br J Dermatol. 2011 Nov;165(5):1011–21. doi: 10.1111/j.1365-2133.2011.10536.x (PMC3202027; doi:10.1111/j.1365-2133.2011.10536.x)
Supplement: Supplementary file 1 [file bjd0165-1011-SD1.doc]

**Supplementary Information**

**Supplementary Materials and Methods**

***Supplementary statistical analysis*.** The following sun exposure measures were calculated: total daily sun exposure (hrs/day), total weekday sun exposure (hrs/day) overall and separately for the warmer and cooler months, total weekend sun exposure (hrs/day) overall and separately for the warmer and cooler months, total sunny holiday exposure (hrs/year), total sunny holiday exposure between 10am and 2pm (hrs/year), total sunny holiday exposure below 45°N latitude (hrs/year) and total sunny holiday exposure below 45° N latitude between 10am and 2pm (hrs/year). These sun exposure variables were then averaged by age at recruitment. The number of significant sunburns during the warmer months before and at or after the age of 20 was calculated, and the total number of sunburns during lifetime was averaged by age at recruitment. A scoring system was implemented for sunscreen use based on the frequency of usage during the warmer months and was categorized into approximate tertiles describing: “Never or hardly ever”, “Not often”, “Often”. Similarly, sun protection factor (SPF) level was grouped as: “Never or hardly ever used sunscreen”, “Used a SPF < 10”, “Used a SPF ≥ 10”. Sunbed use was dichotomised as ever/never used and also quantified as the number of sunbed sessions during life.

In order to derive a proxy, cumulative measure for sun sensitivity, factor analysis was applied to six correlated phenotypic variables: hair colour, eye colour, self reported freckles as a child, propensity to burn, ability to tan and skin colour on the inside upper arm. Multivariate imputation was used to impute incomplete data for the factor analysis. The estimated first factor scores were averaged over the five imputation sets; the average was used as a proxy for sun sensitivity. The median score was used as the cut-off point to partition participants into sun sensitive and non sun sensitive phenotypes.

*MC1R* genotypes were considered singly, according to the allelic variant classification in “R” and “r”[^1^](#_ENREF_1) and were also grouped into three categories: “Wild type”, “Presence of small *r* variants without a big *R* variant” and “Presence of at least one big *R* variant”. BMI was categorized as “normal or under weight” (<25), “overweight” (25-29.99) and “obese” (≥30) according to the “WHO Expert Consultation’s International Classification of adult underweight, overweight and obesity”[^2^](#_ENREF_2). Smoking was dichotomised as ever/never smoked, and also quantified as pack year (number of daily cigarettes*years smoked/20). The average number of alcohol units (wine, beer and spirits) consumed per week was calculated. Highest educational level attained was grouped as “Primary/Secondary school” “Sixth form/Vocational training” and “Univerity/Post graduate”.

Since phenotype was measured on an ordinal scale, proportional odds regression models were used to determine predictors of wrinkles, vascularity and pigmentation, which were interpreted as follows. The odds ratio (OR) of 7.52 for males versus females for vascularity (Table 3), for example, implies that whatever cut-off is chosen, the odds of being in the higher versus lower category are 7.52 times greater for a man than for a woman, holding other variables constant. If a vascularity score of ≥2 versus <2 is considered, the crude OR estimate for males versus females is 6.3, for a cut-off of 3 the OR is 7.1 and for a cut-off of 4 the OR is 10.4. The proportional odds model has the effect of smoothing these estimates to derive the overall estimate of 7.52.

The parallel regression assumption of proportional odds regression was tested using a Wald-type test[^3^](#_ENREF_3). The only covariate showing evidence of violation of this assumption was age, when assessed as a predictor of wrinkles and vascularity (but not pigmentation). Further analysis using multinomial logistic regression showed that, in relation to baseline scores, age was a disproportionately greater risk factor for the most severe category of both wrinkles (score >5) and vascularity (score 4) when compared to the effects in the other, less severe, categories. The proportional odds regression analyses adjusting for all factors other than age were repeated. These models conformed to the parallel regression assumption, and there was minimal change in the ORs for the other covariates.

**Supplementary References**

1 Duffy DL, Box NF, Chen W *et al.* Interactive effects of MC1R and OCA2 on melanoma risk phenotypes. *Hum Mol Genet* 2004; **13**: 447-61.

2 WHO. Obesity: preventing and managing the global epidemic. Report of a WHO consultation. *World Health Organ Tech Rep Ser* 2000; **894**: i-xii, 1-253.

3 Brant R. Assessing proportionality in the proportional odds model for ordinal logistic regression. *Biometrics* 1990; **46**: 1171-8.

**Supplementary Tables**

**Table S1.** Predictors of the three skin aging measures in proportional odds regression models found to be significant at the 5% level in the primary analysis, stratified by melanoma status.

|  | **Combined estimate^1^** | **Cases**  **(797)^2^** | **Controls**  **(441)^2^** |
| --- | --- | --- | --- |
| **Wrinkles** | | | |
| **Age at examination^3^** | **2.50 (2.26-2.76)** | 2.61 (2.30-2.97) | 2.31 (1.98-2.70) |
| **Sex (Male vs Female)^3^** | **1.56 (1.28-1.89)** | 1.32 (1.02-1.70) | 2.06 (1.51-2.81) |
| **Highest educational level^3^** |  |  |  |
| Primary/Secondary School | **1** | 1 | 1 |
| Sixth Form/Vocational Training | **0.71 (0.57-0.88)** | 0.68 (0.52-0.91) | 0.82 (0.58-1.17) |
| University/Post-graduate | **0.55 (0.42-0.72)** | 0.52 (0.37-0.74) | 0.59 (0.40-0.89) |
| **Sunscreen - SPF level** | **0.84 (0.74-0.96)** | 0.87 (0.73-1.03) | 0.83 (0.67-1.02) |
| **Vascularity** | | | |
| **Age at examination^3^** | **1.35 (1.23-1.48)** | 1.33 (1.19-1.48) | 1.38 (1.19-1.59) |
| **Sex (Male vs Female)^3^** | **7.52 (5.99-9.45)** | 7.74 (5.74-10.43) | 7.28 (5.10-10.39) |
| ***MC1R* genotype** |  |  |  |
| wild type | **1** | 1 | 1 |
| r/wt | **1.39 (0.99-1.94)** | 1.07 (0.69-1.68) | 1.99 (1.19-3.32) |
| r/r | **1.25 (0.80-1.96)** | 0.77 (0.42-1.40) | 2.40 (1.21-4.76) |
| R/wt | **1.09 (0.78-1.52)** | 0.83 (0.54-1.28) | 1.60 (0.95-2.71) |
| R/r | **1.45 (1.01-2.10)** | 0.96 (0.62-1.51) | 3.30 (1.70-6.39) |
| R/R | **1.73 (1.10-2.72)** | 1.23 (0.74-2.05) | 5.02 (1.47-17.10) |
| ***MC1R* genotype – grouped** |  |  |  |
| wild type | **1** | 1 | 1 |
| “r” without “R” | **1.35 (0.99-1.85)** | 0.96 (0.64-1.46) | 2.10 (1.30-3.41) |
| “R” | **1.29 (0.97-1.73)** | 0.96 (0.66-1.39) | 2.15 (1.32-3.48) |
| **BMI** | **1.23 (1.07-1.41)** | 1.19 (1.00-1.43) | 1.24 (0.99-1.55) |
| **Pack year^4^** | **1.21 (1.02-1.43)** | 1.21 (1.02-1.43) | - |
| **Average daily sun exposure** | **1.12 (1.02-1.24)** | 1.11 (0.98-1.27) | 1.12 (0.96-1.30) |
| **Average weekday sun exposure – Overall** | **1.12 (1.02-1.23)** | 1.11 (0.99-1.26) | 1.13 (0.97-1.31) |
| **Average weekday sun exposure - Cooler months** | **1.11 (1.01-1.22)** | 1.12 (0.99-1.27) | 1.09 (0.93-1.26) |
| **Pigmentation** | | | |
| **Age at examination^3^** | **1.12 (1.03-1.23)** | 1.08 (0.97-1.21) | 1.17 (1.01-1.35) |
| **Sex (Male vs Female)^3^** | **1.43 (1.17-1.74)** | 1.35 (1.04-1.75) | 1.55 (1.13-2.13) |
| **Highest educational level^3^** |  |  |  |
| Primary/Secondary School | **1** | 1 | 1 |
| Sixth Form/Vocational Training | **1.29 (1.03-1.62)** | 1.29 (0.97-1.72) | 1.28 (0.89-1.85) |
| University/Post-graduate | **1.57 (1.20-2.05)** | 1.64 (1.14-2.35) | 1.58 (1.05-2.37) |
| ***MC1R* genotype** |  |  |  |
| wild type | **1** | 1 | 1 |
| r/wt | **0.77 (0.56-1.07)** | 0.76 (0.49-1.18) | 0.78 (0.47-1.29) |
| r/r | **0.56 (0.36-0.86)** | 0.60 (0.34-1.07) | 0.48 (0.24-0.96) |
| R/wt | **0.55 (0.39-0.76)** | 0.60 (0.40-0.92) | 0.46 (0.27-0.77) |
| R/r | **0.58 (0.41-0.83)** | 0.65 (0.42-1.01) | 0.44 (0.23-0.84) |
| R/R | **1.15 (0.72-1.83)** | 1.27 (0.76-2.13) | 0.63 (0.18-2.12) |
| ***MC1R* genotype – grouped** |  |  |  |
| wild type | **1** | 1 | 1 |
| “r” without “R” | **0.71 (0.52-0.96)** | 0.71 (0.47-1.07) | 0.71 (0.44-1.14) |
| “R” | **0.63 (0.47-0.83)** | 0.73 (0.51-1.05) | 0.47 (0.29-0.76) |
| **Average weekday sun exposure - Warmer months** | **1.11 (1.01-1.22)** | 1.09 (0.97-1.23) | 1.14 (0.98-1.32) |
| **Sunscreen - Used to stay in the sun longer** | **0.86 (0.76-0.98)** | 0.85 (0.72-1.01) | 0.89 (0.72-1.10) |

^1^ Adjusted for age, sex, education and melanoma status, unless otherwise stated.

^2^ Adjusted for age, sex and education, unless otherwise stated. Not every model included the total, due to missing data.

^3^ The combined estimate is adjusted for melanoma status. The stratified estimates are unadjusted.

^4^ Smoking history data were collected for melanoma cases only. Adjusted for age, sex and education.
